# A Singular Value Decomposition based Maximal Poisson-disk Sampling for adaptive Digital Elevation Model simplification

**DOI:** 10.1371/journal.pone.0238294

**Published:** 2020-09-01

**Authors:** Xingquan Wu, Zhiwei Li, Hongyuan Zhang, Xin Li, Wenguang Hou, Xiaofeng Ma

**Affiliations:** 1 China Energy Engineering Group Gansu Electronic Power Design Institute Co. Ltd, Lanzhou, China; 2 College of Life Science and Technology, Huazhong University of Science and Technology, Wuhan, China; 3 Beijing North-Star Digital Remote Sensing Technology Co. Ltd, Beijing, China; Utrecht University, NETHERLANDS

## Abstract

The proposed method is to do simplification for Digital Elevation Model (DEM), which uses a few of original nodes representing the terrain surface while maintaining the accuracy. The original DEM nodes are sampled using the Maximal Poisson-disk Sampling (MPS), in which, the disk’s size of each sample is computed on basis of the Singular Value Decomposition (SVD). MPS can generate the hyper-uniformly distributed samples and was taken to do DEM adaptive sampling by being combined with the geodesic metric. However, the geodesic distance computation is complex and the requirement for memory is high. As such, this paper proposes an extension of the classic MPS based method for selecting quasi-randomly distributed points from DEM nodes based on the distribution of eigenvalues, accounting for surface heterogeneity. To achieve this objective, uniform MPS is conducted to sample the DEM nodes by setting the related disk radius to be inversely proportional to the local terrain complexity, which is defined as an index expressing the local terrain variation. Then, the geodesic metric related parameters are implicitly contained in the defined index. As a result, more samples are concentrated in the rugged regions, and vice versa. The proposed method shows better perfermance, at least the results are comparable with the geodesic distance based Poisson disk sampling method. Meanwhile, it greatly accelerates the sampling process and reduces the memory cost.

## Introduction

With the development of Photogrammetry [1], remote sensing and laser scanning, it is easy for us to obtain denser points from the terrain surface with high accuracy [2]. As such, the generated Digital Elevation Model (DEM) usually has high resolution. The high accurate DEM is theoretically helpful in many applications, yet it greatly increases the computation and demands lots of memory in data processing. In many applications, however, the high resolution DEM will not lead to a meaningful improvement. For example, the DEM with 1m or even lower resolution can meet most of our demands about the map navigation in daily life [3]. It means that the value of higher resolution DEM is minor in this case. Therefore, how to choose a certain number of data from the original dense points, which can effectively retain the details of the surface, should be an important issue in practical applications. This paper intends to obtain quasi-randomly distributed points on a surface, accounting for surface heterogeneity leading to high accuracy reconstruction. To achieve this aim, the classic Maximal Poisson-disk Sampling (MPS) is taken here and the spatial structure of the surface is estimated via Singular Value Decomposition (SVD). It is out of the consideration that the eigenvalues’ distribution of each patch (square subregion) is highly related to its terrain complexity. The terrain complexity should be proportional to the sampling rate in adaptive sampling. As such, our contribution lies in that it replaces the geodesic distance using the estimated index from the eigenvalues through SVD. The estimated index is taken to establish the sampling distance. The method can obtain the comparable results with the classic Poisson disk sampling. Yet, the computation is greatly reduced.

## Related works

The DEM, a typical raster data, can be regarded as an image where its planar coordinates and its height are considered as the position of the image pixel and the corresponding gray value, respectively [1]. As such, many DEM processing algorithms were borrowed from image processing. The commonly used way for image simplification is to generate the pyramid layers of the image on basis of the traditional regular sampling methods including bilinear and bicubic algorithms [4]. This way is simple and the compression rate is high. However, it equally reduces the image’s details without considering the distribution of the image structure. As such, the samples contain less detail of the original image. Moreover, the image reconstructed from the regular samples shows aliasing, i.e., the signals are overlapped leading to blurry effect. By comparison, there is no aliasing in random sampling. Yet, the samples’ distribution of random sampling is visually uncomfortable and the reconstruction accuracy is still low. The Farthest Point Strategy (FPS) obtained high sampling accuracy and fine visual effect by gradually sampling the point which is farthest from the accepted samples. It is based on the fact that the sampling pattern that gives the best visually pleasing result should have a blue spectrum, i.e., there is no power peak at any nonzero frequency on its power spectrum [5, 6]. For the blue noise sampling, most of the noise power is concentrated at the high-frequency range, to which the human eye is less sensitive. In case of the regular sampling, some of noise power appears at the low-frequency leading to aliasing in the reconstructed image.

The MPS and FPS have the blue power spectrum and can generate uniform and irregular samples. However, in many applications, the adaptive samples are needed which means more samples should concentrate in the regions with details, and less samples will appear in the smooth parts. To achive this aim, a weighted distance function was defined to move the farthest point to a new location where the value of the raster cell has dramatic change in [5]. The new location should be in the neighbor of the farthest point. After then, the sampling process is progressively performed till the samples’ number reaches the demand. Meanwhile, this paper pointed out that the most natural augmentation to the FPS would be to define a different metric expressing the structure of the image and to choose the point which is the farthest in this metric [5]. For example, the image can be seen as a 2D manifold surface when the value of the raster cell is considered as the height of each pixel. Then, the [7] defined the geodesic metric to express the terrain surface and then applied the MPS in the new metric to obtain the adaptive samples in the Euclidean metric. It is out of the consideraten that the computation of FPS is more complex than that of MPS in sampling. In the implementation, the method randomly selects a point from original nodes and then judges whether this point can be accepted or not in accordance with the related geodesic distances from the sampled points. The sampling process is repeated until no more points can be accepted. The difference between the reconstructed DEM using the adaptive samples and original DEM is small. Besides, the samples’ distribution is well visualized. Theoretically, the geodesic distance computation is a global search problem since the geodesic distance between two points should be the length of the shortest way from the starting point to the target taking other points in neighbors as midpoints. On basis of the greedy algorithm, fast marching was taken to compute the geodesic distance [8]. However, its computational burden is substantial [7].

As such, this paper still restricts the computation in the Euclidean metric. Our motivation is to propose a new way to adaptively estimate the radius in MPS rather than finding the points whose geodesic distances to the considered sample are less than the radius, i.e., we make a change for the classic MPS in Euclidean metric. Moreover, the proposed method is compared with the algorithm in [7] considering that it is classic, effective and robust in the two dimensional manifold sampling such as the DEM. To reach this aim, SVD will be taken here to estimate the disk’s radius for each sampled node in the process of MPS. Qualitatively, the disk’s size is small when the sampled point is located in the rugged region and it should be big in the reverse case. It results in the situation that more points are sampled in the rugged regions and vice versa.

## Methodology

### The basic idea

**Fig 1:**
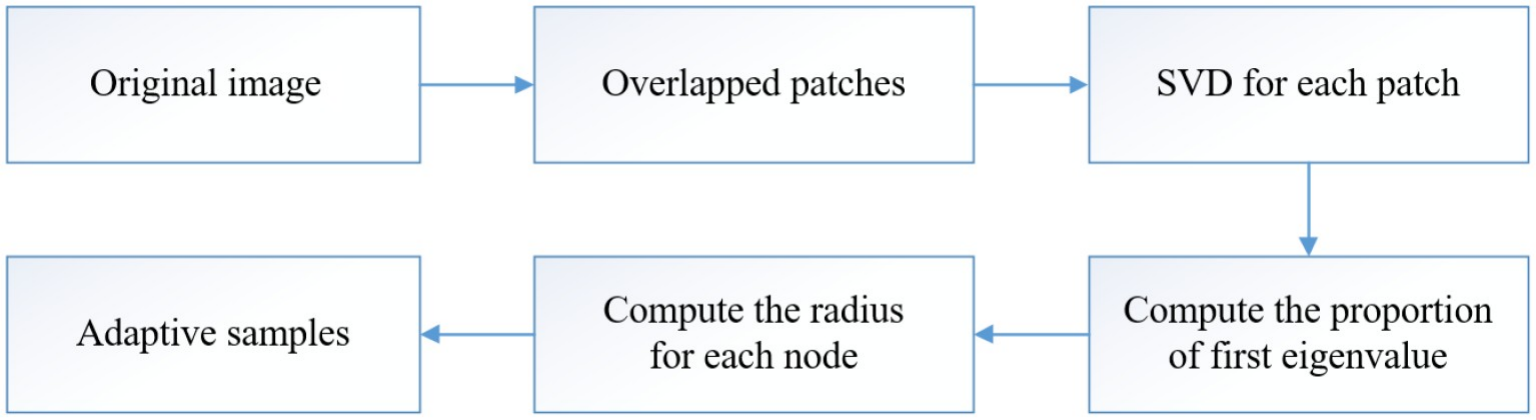
The flowchart of SVD based maximal Poisson-disk sampling for DEM simplification.

**Fig 2:**
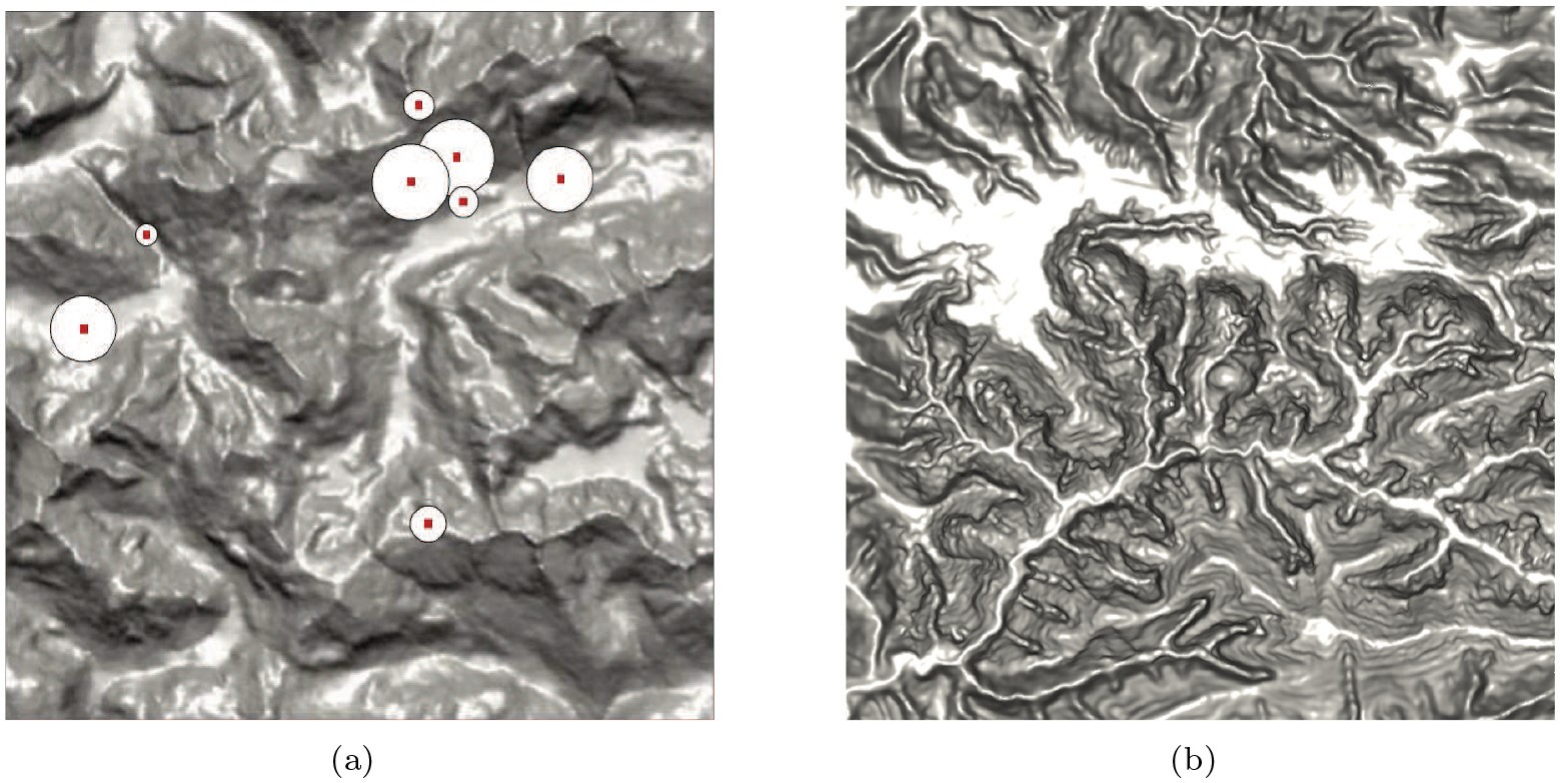
The principle of SVD based maximal Poisson-disk sampling for DEM simplification is shown in (a). The center of each disk is the final sample, which randomly appears at the DEM node. The disk’s radius is related to the local terrain variation. Moreover, one sample is only located in its related disk. Meanwhile, (a) and (b) are two data used in the following experiment.

Taking the raster DEM into account, our adaptive DEM simplification method includes two main steps. The first is to compute an index to denote the terrain complexity for each node. Then we perform MPS for all nodes, where the radius for each node is roughly inverse to the estimated terrain complexity index. Fig 1 shows the main flowchart, in which, the third to fifth steps are taken to compute the terrain complexity index and the last step perform adaptive MPS. Fig 2 is our principle. In sampling, each disk (circle) is randomly distributed on the DEM surface and its center will be the sample. It should be mentioned that each disk only contains one sample. Moreover, the radii of different disks are different shown as Fig 2(a). In the rugged regions, the radii are small and vice versa. The sampling process is repeated till the whole DEM is covered by the disks. It means that no more space can accommodate any sample. According to this rule, more samples will concentrate in the rugged regions and less samples should appear in the flat parts. The radius of each disk is computed on the basis of the eigenvalues of the window centred at the considered node through SVD [9]. Accordingly, the more uneven the eigenvalues are, more details are contained in the patch. More details mean that the considered point should be sampled with more chances. It is similar in the reverse situation. As such, we can obtain an index for each node to denote its local terrain complexity which should be inversely proportional to the radius of circle in Fig 2. In the second step, the dart throwing algorithm is applied to perform MPS, in which, the radii of different samples are different and the samples are randomly distributed. Finally, the trade-off between uniformity and adaptivity can be obtained in the sampling results.

### Maximal Poisson-disk Sampling

**Fig 3:**
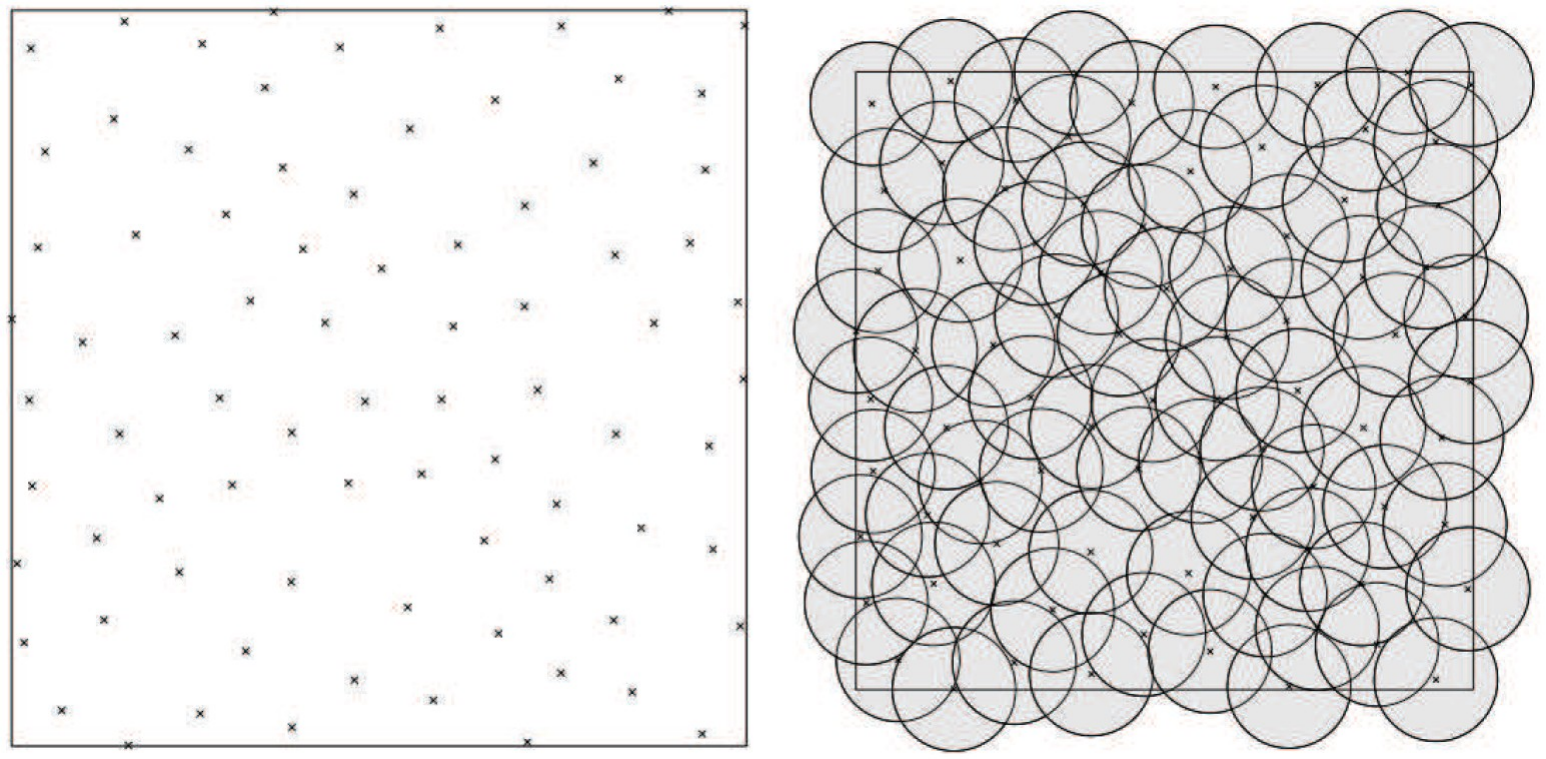
The principle of maximal Poisson-disk sampling [13]. The samples are hyper-uniformly distributed. The distance between any two samples is larger than the largest radius of the disks.

In MPS, the distance between any two samples is larger than a threshold. Moreover, no more points can be inserted into these samples, i.e., adding one more point in the samples will break the rule that the distance between any two samples is longer than the given threshold [10–12]. Its principle is shown in Fig 3, the distance of any two samples is more than the radius of disk and the disk is randomly distributed which results in the uniformly and randomly distributied samples [13, 14]. As a classic Poisson disk sampling method, the dart throwing method randomly generates a point and the point will be accepted when its distances to other accepted samples are smaller than the threshold. If not, a new point will be generated and then judged again [15, 16]. In uniform sampling, the radii of all disks are same. However, many applications prefer to obtain the adaptive samples. Then, the radii should be different in different parts [14]. Besides, the FPS can obtain the samples with similar spatial distribution as that of MPS. Yet, its computation is much higher. Moreover, the difficulty increases when the non-Euclidean metric is considered [5]. As such, MPS is widely used.

### Singular Value Decomposition

The singular value decomposition of matrix *B* is as follows:

$$\begin{array}{c} B=U\sqrt{\Lambda }V. \end{array}$$ (1)

Any real matrix can be represented as a product of Eq (1), where *U*, *V* are unitary matrices and $\sqrt{\Lambda }$ is a diagonal matrix with non-negative entries. The Eq (1) can then be transformed into Eq (2), in which, λ_*i*_ is the i-th eigenvalue, *u*_*i*_ and *v*_*i*_ are the related eigenvector in matrix *U* and *V*. Generally, {λ_*i*_} are in descending order.

$$\begin{array}{c} B=\sum \lambda_{i}u_{i}v_{i}^{T} \end{array}$$ (2)

From Eq (2), we can obtain different Level-of-Detail (LOD) data when considering different numbers of eigenvalues and the related eigenvectors. Furthermore, some eigenvalues are close to zero, which means that their effect in reconstructing matrix *B* is minor [17]. Then, these eigenvalues and the related eigenvectors can be ignored. Because SVD can obtain the eigenvalues that represent the structure of the original data, it has been widely used in many applications including image processing and pattern recognition [18, 19].

### Capturing spatial structure via SVD

SVD in conducted on the patch centred at each node of the raster DEM. Suppose that the size of patch is *m*. We then can obtain the related eigenvalues. It is common that the eigenvalues $\lambda_{1}^{i},\lambda_{2}^{i},\ldots ,\lambda_{m}^{i}$ are in descending order. Moreover, $\lambda_{1}^{i}$ should be much bigger than $\lambda_{2}^{i},\ldots ,\lambda_{m}^{i}$ in most cases. In other words, $\lambda_{2}^{i},\ldots ,\lambda_{m}^{i}$ can be neglected when there are fewer details in the considered window, i.e., their effect in reconstructing the original matrix is minor. Thus, the following equation can be used to estimate the amount of details.

$$\begin{array}{c} s_{i}=\frac{\lambda_{1}^{i}}{\sum_{j=1}^{m}\lambda_{j}^{i}} \end{array}$$ (3)

In Eq (3), *i* refers to the number of the considered patch, *j* denotes the eigenvalue’s number and *m* represents the size of patch. It is obvious that the higher that *s*_*i*_ is, the fewer details the considered patch has. Similarly, for the nodes in the rugged patches, the related *s*_*i*_ should be small. As such, *s*_*i*_ is an effective index to express the spatial structure of DEM.

### Computing the radius for each node

According to our idea, the radius of each node in MPS should be proportional to the *s*_*i*_. In this article, the radius is computed as follows. Firstly, we sort the {*s*_*i*_} in ascending order. Then, {*s*_*i*_} are evenly divided into several sets {*T*_*j*_}. For all nodes in each set, the related radii are same. It is clear that the radii of {*T*_*j*_} are decreasing as *j* rises. The size of patches in capturing spatial structure is advised to be more than 7 * 7 and the number of sets should be more than 3. Moreover, the minimum radius is set to 2. These parameters can generally lead to fine visual effect and high reconstruction accuracy. It should be mentioned that this is just an example. Specific application needs to choose specfic parameters. After computing the radius of each node, we will perform MPS on the image / DEM. The difference from the uniform sampling lies in the radius of each node is different.

## Experiment and result

As above mentioned, the raster DEM can be seen as an image. Then, we will conduct the sampling for the image and DEM in this section. The sampling results are visually judged and quantitatively evaluated by comparing the reconstructed surface with the original one. The experiments show that the proposed method can obtain adaptive samples with high accuracy. In addition, the algorithm is simple and the computation is moderate. The data in this experiment can be found in [20].

### Image sampling

**Fig 4:**
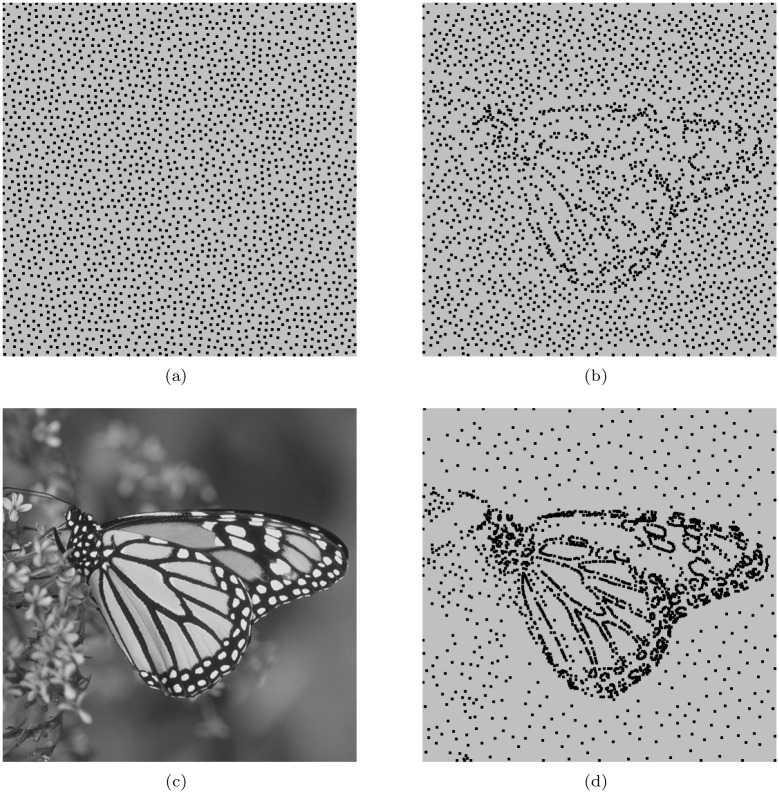
The samples in different cases. (a) shows the uniform samples, (b) is the result in definition of geodesic distance and (d) is one in weighted geodesic distance case. (c) is the image of *Butterfly*. Compared with (a) and (b), (d) obtains the adaptivity.

We first perform the uniform sampling for the image and DEM on basis of the dart throwing-based MPS. The *Butterfly* image [20] with 512 × 512 cells, is used here. It is shown in Fig 4(c). We firstly conduct uniform sampling for the nodes. The radius of the disk is set to 9, i.e., the Euclidean distance between any two final samples is longer than 9. It is clear that less samples will be obtained when the radius of the disk increases, and vice versa. The size of radius depends on the specific application. 9 is just an example for illustraten. Meanwhile, the contrast experiment is done where the geodesic distance between any samples is set to 16. The geodesic distance between two neighbor points is calculated by Eq (4), in which, *x*_*i*_, *y*_*i*_ are the planar coordinates, the *h*_*i*_ is the value of each node and *c* = 1. For any two points, their geodesic distance is estimated by adding the Euclidean distances between two midpoints along the geodesic line from the start to target. The geodesic line means that the computed distance along the line is the smallest. In addition, the 4-nearest neighbors are considered in finding the geodesic line, which denotes that neighbors of the considered point are the four points whose planar distances to the current point are less than others [7].

$$\begin{array}{c} G_{i}^{j}=\sqrt{{\left(x_{i}-x_{j}\right)}^{2}+{\left(y_{i}-y_{j}\right)}^{2}+c\cdot {\left(h_{i}-h_{j}\right)}^{2}}, \end{array}$$ (4)

It is clear that for any two points, their geodesic distance should be longer than (at least equal to) their related Euclidean distance. As such, the number of samples in the definition of Euclidean distance should be less than that of geodesic distance case when the disks’ radii are same. To obtain the same number of samples, we rise the radius of disk in geodesic metric sampling as above mentioned. Fig 4(a) shows hyper-uniformly distributed samples according to the Poisson disk model in Euclidean metric. In case of the geodesic distance, the result is shown in Fig 4(b). By comparison, the samples in Fig 4(b) are hyper-uniformly distributed in the geodesic metric rather than Euclidean metric. In addition, when we want to obtain more adaptive samples, the geodesic distance between two neighboring points will be replaced by the weighted one, i.e., *c* > 1. It means that we emphasis the height difference between two neighbor nodes in computing their distance. As such, the weighted geodesic distance between any two points will be more than that of original one. Yet, the weighted one between points in rugged parts increases rapidly. It then makes more samples gather in the rugged regions. Fig 4(d) shows the samples in case of weighted geodesic distance. According to them, adaptive sampling can be obtained by combining MPS and the weighted geodesic distance.

### DEM sampling

**Fig 5:**
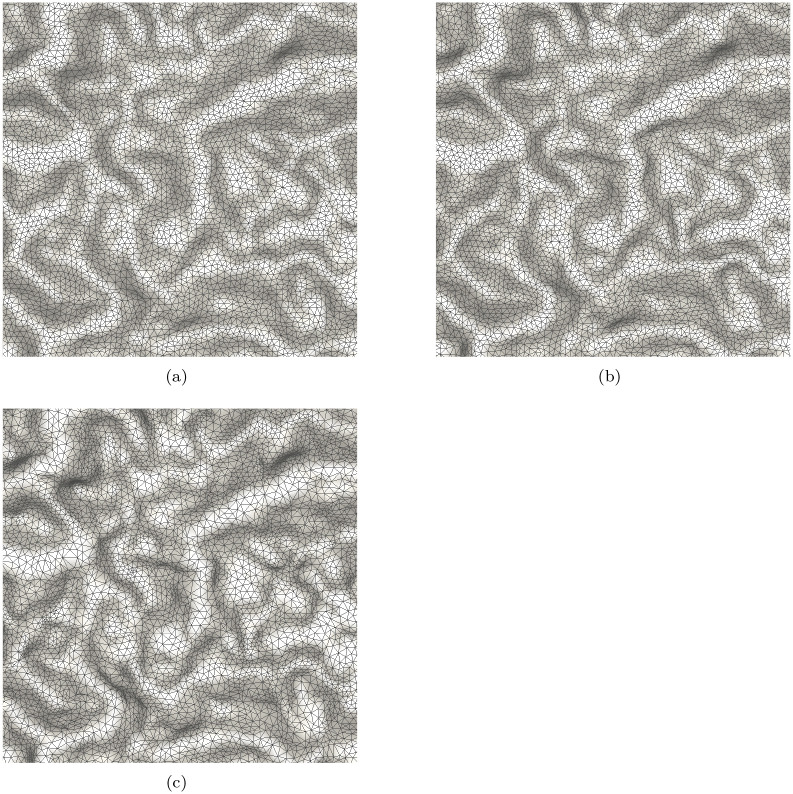
The sampling results of a DEM on basis of MPS. (a), (b) and (c) correspond to the cases of Euclidean distance, geodesic distance and the weighted geodesic distance. It should be mentioned that the numbers of samples are same for three cases.

We conduct DEM sampling using the same parameters as the above experiment. The original data is shown in Fig 2(a). The testing DEM also has 512 × 512 nodes. The sampling results are shown in Fig 5, in which the samples are connected by Triangular Irregular Networks (TIN). It can be found that the difference between the uniform and the geodesic distance based sampling results is not obvious as Fig 5(a) and 5(b). This is because the DEM is smoother than the *Butterfly* image. By comparison, the adaptive sampling result in Fig 5(c) shows a good balance between uniformity and adaptivity. For Poisson disk sampling, the number of samples may have small variations in different tests even all the parameters are same. As such, we perform the Poisson disk sampling tests for 10 times in each case. The average number is taken as the final sampling number. In addition, the cost of geodesic based uniform sampling is 10 times that of Euclidean-based sampling.

**Fig 6:**
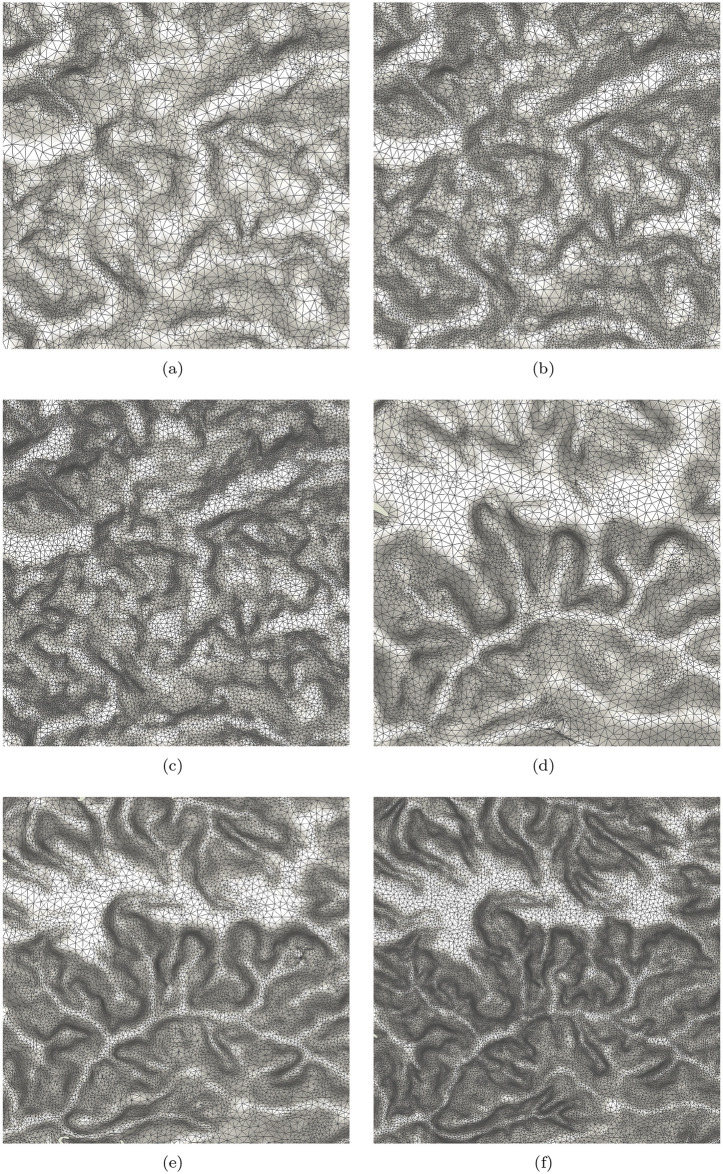
The sampling results of two data through MPS on basis of the eigenvalues from SVD. The adaptive sampling rates are (a): 2%, (b): 5% and (c): 10%, respectively. (d), (e) and (f) are the corresponding results of another data.

**Fig 7:**
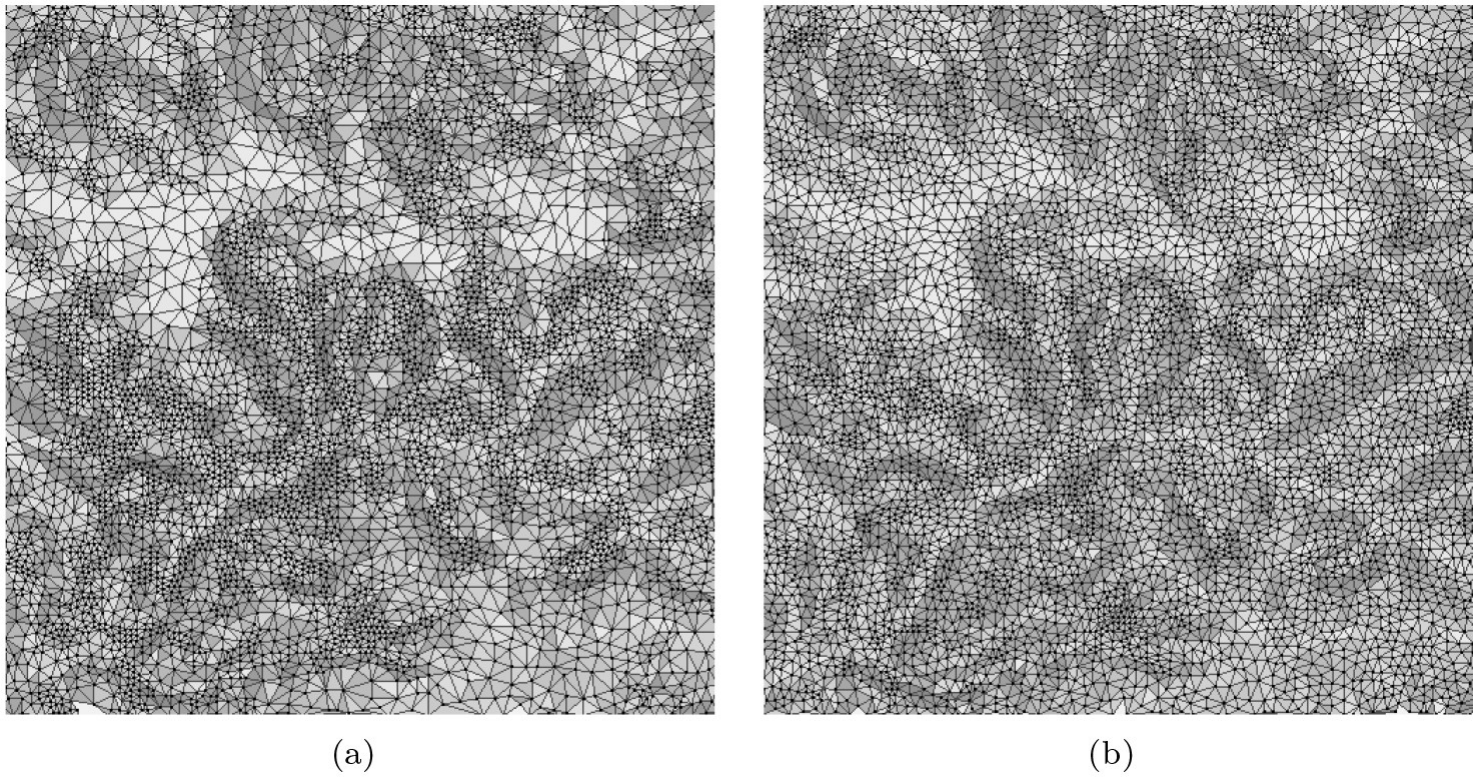
The mesh models of the samples obtained from the low resolution version of the Fig 2(b).

We conduct adaptive sampling on basis of the proposed method. Firstly, the patch with 11 × 11 cells centred at each node is detected from the DEM. SVD is then conducted for all patches. As such, a 11-dimension eigenvalue vector for each node can be obtained. For each node, the proportion of the first eigenvalue to all eigenvalues can be computed as *s*_*i*_ according to Eq (3). The max and min *s*_*i*_ are 0.999 and 0.996 in this experiment. The {*s*_*i*_} is sorted in ascending order and is evenly divided into five sets. For the node in each set, we calculate a corresponding radius. After obtaining the radii for all nodes, the MPS is applied to do sampling, in which any two samples’ distance is lager than the related largest radius. Fig 2(b) is the original data and Fig 6 shows the sampling results. For 6(a) and (d), the radii are {3, 5, 7, 9, 11}. They are {3, 4, 6, 8, 9} and {2, 3, 5, 7, 9} for Fig 6(b), 6(e), 6(c) and 6(f), respectively. The corresponding sampling rates are about 2%, 5% and 10%. To validate the effectiveness of our method in multi level cases, we downsample the Fig 2(b) through interval sampling, i.e., the size of the low resolution image is 1/4 of its original one. Then, adaptive sampling are done for the degenerate image. Fig 7(a) and 7(b) show the results. The parameters in generating Fig 7(a) are same as above. The *s*_*i*_ are evenly divided into three sections in generating the Fig 7(b). Compared them with the Fig 6, many details are missing. Yet, the main features are still reserved which shows that the method is robust in multi level cases.

**Fig 8:**
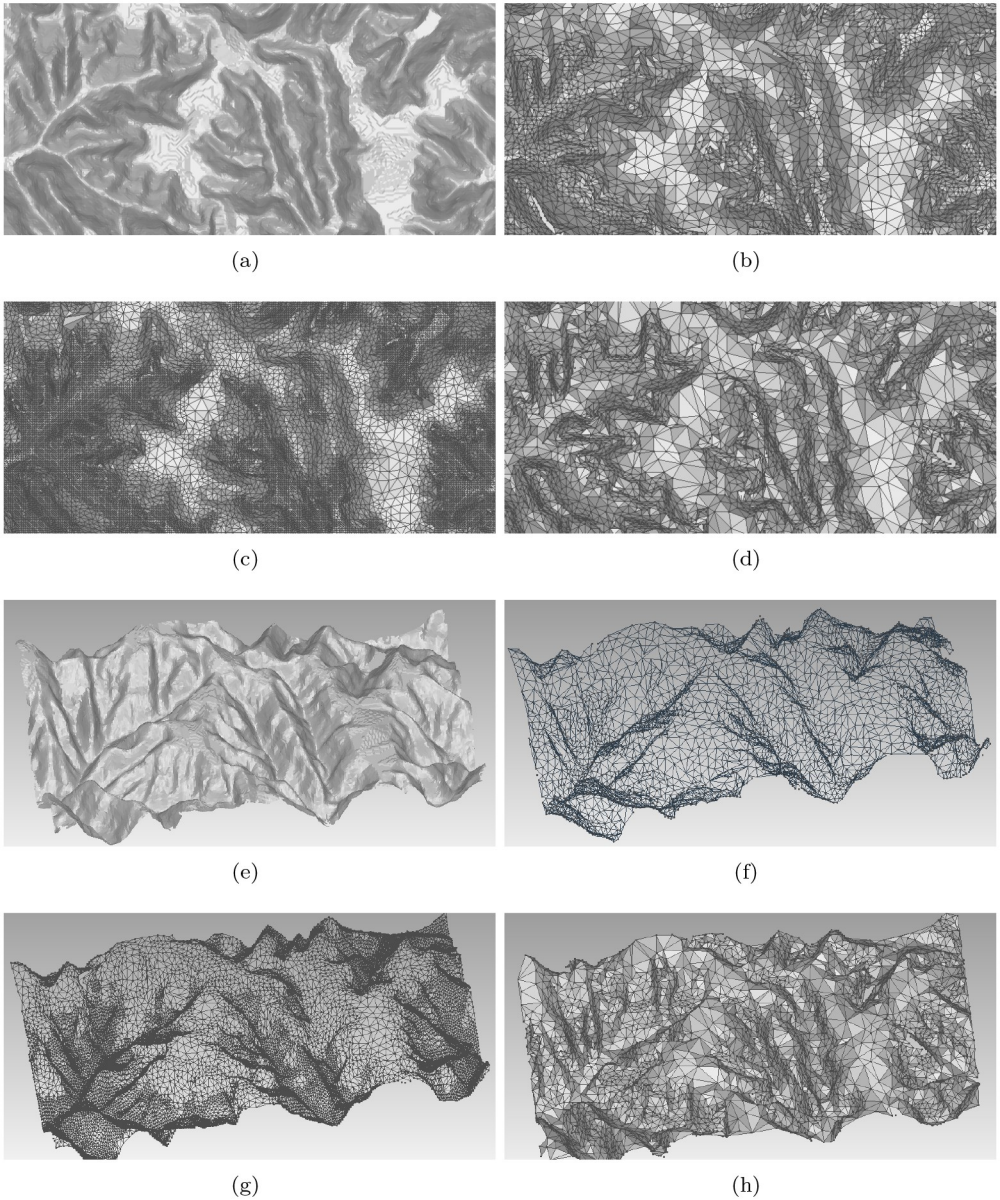
The reconstruction surfaces and mesh models. (a) is the original surface of a rugged DEM with steep slope; (b), (c) are the reconstructed surface using the 10% and 50% samples obtained by our method; (d) is the result of Poisson disk sampling; (e)-(h) are the mesh models corresponding to (a)-(d). Samples trade off the uniformity and adaptivity.

Meanwhile, a mountain DEM is taken to test the proposed method shown in Fig 8. Fig 8(a) is the original DEM surface, Fig 8(b) and 8(c) are the reconstructed surfaces using 10% and 50% samples obtained by SVD based method, Fig 8(d) is the result of Poisson disk sampling. Fig 8(e)–8(h) are the non orthophoto mesh models of Fig 8(a)–8(d). For this data, the max and min *s*_*i*_ are 0.999 and 0.825 since the data is obviously rugged. Similarly, we divide the {*s*_*i*_} into subsets and compute the related radius in MPS. According to them, more details appear in the reconstruction with the increase of the sampling rate. However, the details are mostly retained in the low sampling cases. Moreover, the sample distribution achieves a balance between being uniformity and adaptivity for SVD and Poisson disk sampling methods. It should be mentioned that the former DEM is relatively smooth and the latter one has many sharp changes. Though that, the proposed method can obtain fine results. It shows that the proposed method is robust in cases of rugged and plain DEMs.

### Error quantification

**Fig 9:**
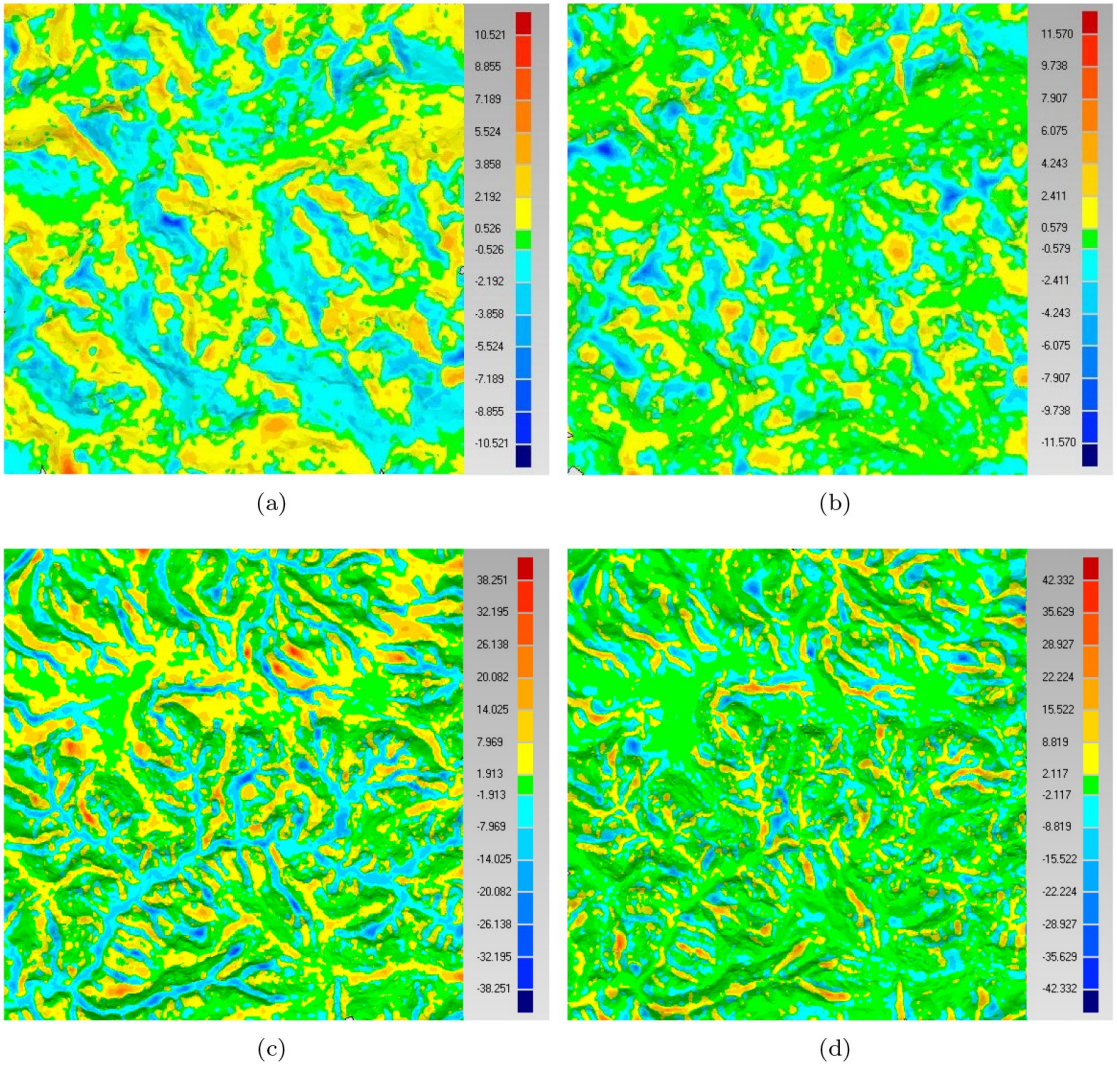
The reconstruction error distribution. (a) is the reconstruction error of geodesic distance based maximal Poisson-disk sampling, (b) is our result. Correspondingly, (c) and (d) are the reconstruction errors of data 1.

**Fig 10:**
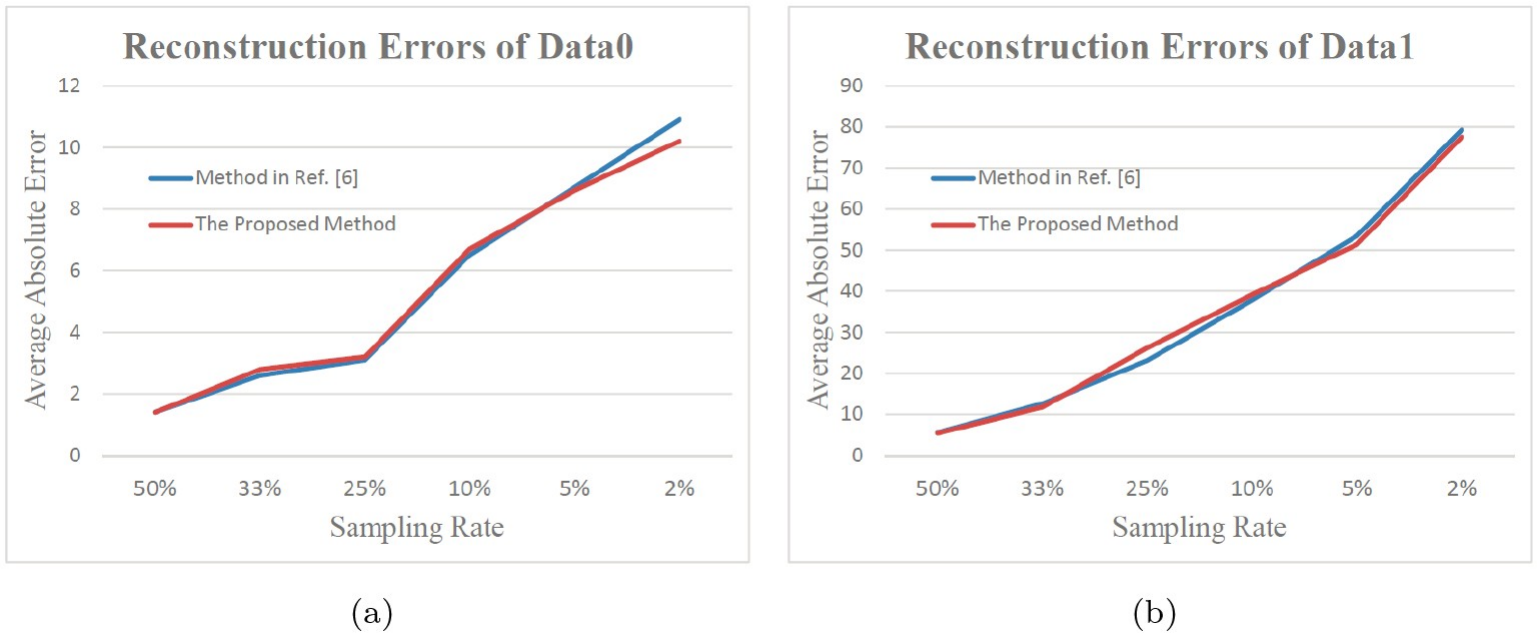
Comarison with the classic method of [7]. (a) shows the reconstruction errors of Data0 in cases of different sampling rates, (b) is related result of Data1. According to them, the accuracy of SVD based method is very close to and a little higher than that of Geodesic based method in [7]. Yet, its computation efficiency is about five times of the latter.

In addition, we quantitatively evaluate the sampling accuracy. Firstly, we reconstruct the DEM surfaces using the samples in different cases. Specifically, in each case, the mesh model of the samples is generated using the software ‘Geomagic’. Then, we compute the distance of each original DEM node to the mesh model (the reconstructed surface). The distance refers to the shortest length among the all lines from the point to the surface [1, 7]. As such, the average distance of all original points to the reconstructed surface can be obtained for each sampling case. It is clear that the small distance means fine result. Fig 9(a) and 9(b) show the error figures in the cases of the geodesic distance and SVD-based methods, respectively. Fig 9(c) and 9(d) are the results of another dataset. From these error figures, we can see that the SVD-based method pays more attention to the features of the DEM and then reserves more key nodes in the samples. This is especially clear by comparing Fig 9(c) and 9(d). Fig 10 shows the error in detail.

## Conclusion

This paper proposes an SVD-based method that computes the adaptive radius for each node. In the subsequent MPS, the radius of each node is set to be related to the variation of terrain surface. The more rugged the part, the radius is smaller. This way makes more samples appear in the rugged parts. Meanwhile, the samples are the tradeoff between the uniformity and adaptivity. Compared with the classic MPS in geodesic metric, the proposed algorithm is more accurate and the computation is greatly accelerated. In our method, the sampling rate for each node is determined before sampling. Whether a certain node is sampled or not is random. However, the sampling probabilities for the nodes in the rugged regions are higher. As such, the samples are adaptive after Poisson-disk sampling. Overall, the proposed method can obtain higher accuracy and efficiency than the geodesic distance based one. It is clear that the complexity of the manifold is related to the geodesic distance. The precondition that proposed method can perform adaptive sampling in Euclidean metric is that the terrain complexity can be estimated using the eigenvalues. That is, our contribution lies in that the SVD is taken to estimate the manifold related index and then is applied into the application of DEM sampling by combining with the MPS. In addition, the experiments in different cases validate its effectiveness.

In addition, the scale of the DEM need not be condsidered in sampling. There are still some issues to be considered in future. The first is how to perform SVD and Poisson-disk sampling in a parallel way, especially using multi-core central processing unit and graphics processing unit to accelerate the sampling process. According to the proposed method, it could be helpful in capturing rugged regions by scaling the vertical dimension non-linearly. Meanwhile, the uniformity should be attained in the local parts as far as possible. As such, another future work is to find a solution to automatically scale the vertical dimension to obtain the balance between the adaptivity and uniformity.

## Supporting information

S1 Data (RAR)

S2 Data (RAR)

S3 Data (RAR)

S4 Data (RAR)

S5 Data (RAR)
